# CYP450 Mediates Reactive Oxygen Species Production in a Mouse Model of β-Thalassemia through an Increase in 20-HETE Activity

**DOI:** 10.3390/ijms22031106

**Published:** 2021-01-23

**Authors:** Rayan Bou-Fakhredin, Batoul Dia, Hilda E. Ghadieh, Stefano Rivella, Maria Domenica Cappellini, Assaad A. Eid, Ali T. Taher

**Affiliations:** 1 Department of Anatomy, Cell Biology and Physiological Sciences, Faculty of Medicine, American University of Beirut, Beirut 1107 2020, Lebanon; rib05@mail.aub.edu (R.B.-F.); bad02@mail.aub.edu (B.D.); hg36@aub.edu.lb (H.E.G.); 2 Division of Hematology and Oncology, Department of Internal Medicine, American University of Beirut Medical Center, Beirut 1107 2020, Lebanon; 3 Department of Pediatrics, Division of Hematology, The Children’s Hospital of Philadelphia (CHOP), Philadelphia, PA 19104, USA; rivellas@email.chop.edu; 4 Perelman School of Medicine, University of Pennsylvania, Philadelphia, PA 19104, USA; 5 Cell and Molecular Biology Affinity Group (CAMB), University of Pennsylvania, Philadelphia, PA 19104, USA; 6 Raymond G. Perelman Center for Cellular and Molecular Therapeutics-CHOP, Philadelphia, PA 19104, USA; 7 Penn Center for Musculoskeletal Disorders, CHOP, Philadelphia, PA 19104, USA; 8 Fondazione IRCCS Ca’ Granda Ospedale Maggiore Policlinico, Internal Medicine, 20122 Milan, Italy; maria.cappellini@unimi.it; 9 Department of Clinical Sciences and Community Health, University of Milan, 20122 Milan, Italy

**Keywords:** oxidative stress, reactive oxygen species, CYP450, non-transfusion-dependent thalassemia, NADPH oxidases

## Abstract

Oxidative damage by reactive oxygen species (ROS) is one of the main contributors to cell injury and tissue damage in thalassemia patients. Recent studies suggest that ROS generation in non-transfusion-dependent (NTDT) patients occurs as a result of iron overload. Among the different sources of ROS, the nicotinamide adenine dinucleotide phosphate (NADPH) oxidase family of enzymes and cytochrome P450 (CYP450) have been proposed to be major contributors for oxidative stress in several diseases. However, the sources of ROS in patients with NTDT remain poorly understood. In this study, *Hbb^th3/+^* mice, a mouse model for β-thalassemia, were used. These mice exhibit an unchanged or decreased expression of the major NOX isoforms, NOX1, NOX2 and NOX4, when compared to their C57BL/6 control littermates. However, a significant increase in the protein synthesis of CYP4A and CYP4F was observed in the *Hbb^th3/+^* mice when compared to the C57BL/6 control mice. These changes were paralleled by an increased production of 20-hydroxyeicosatetraenoic acid (20-HETE), a CYP4A and CYP4F metabolite. Furthermore, these changes corroborate with onset of ROS production concomitant with liver injury. To our knowledge, this is the first report indicating that CYP450 4A and 4F-induced 20-HETE production mediates reactive oxygen species overgeneration in *Hbb^th3/+^* mice through an NADPH-dependent pathway.

## 1. Introduction

The thalassemias are among the most common groups of recessively inherited disorders worldwide, and are characterized by reduced or absent production of red cell hemoglobin and chronic anemia with varying severity [1,2]. The epidemiology of various forms of the thalassemias remains poorly recognized. However, the disease is vastly prevalent in regions that extend from sub-Saharan Africa, through the Mediterranean region and Middle East, to the Indian subcontinent and East and Southeast Asia [3,4]. Continued and recent population migrations have also meant that the thalassemias can now be found in northern and western Europe and in North America, making this disease a global health concern [5,6,7].

A major transition in the classification of the thalassemias has occurred over the last decade. While the standard and old classification was based on molecular forms, clinicians have moved towards a categorization that is based on clinical-management criteria. Because transfusion therapy is the conventional modality of treatment in patients with thalassemia, the frequency and extent of transfusion requirements indirectly reflect the underlying severity of the disease. The use of blood transfusions in these patients can control most of the underlying pathophysiological mechanisms, and it can also contribute to secondary morbidity [8,9]. Therefore, thalassemia patients today are commonly categorized as having transfusion-dependent thalassemia (TDT) or non-transfusion-dependent thalassemia (NTDT). Patients with TDT commonly present with severe anemia in their early childhood that requires lifelong blood transfusions for survival. NTDT patients, on the other hand, usually present with mild/moderate anemia during their late childhood, or even in adulthood, that only necessitates occasional transfusions in certain clinical settings and for the prevention or management of certain disease manifestations [10]. The hallmarks of this disease are the α- to β-globin chain imbalance leading to ineffective erythropoiesis, chronic hemolytic anemia, and iron overload. Despite being transfusion independent, NTDT patients often experience many clinical complications, mostly due to iron overload. While iron overload in TDT patients is secondary to blood transfusions, iron overload in NTDT patients is due to increased intestinal iron absorption, which is mediated by the hormone hepcidin. Iron overload in NTDT patients is a cumulative process that can lead to significant morbidity (liver, endocrine, vasculature) and mortality [11].

**Figure 1:**
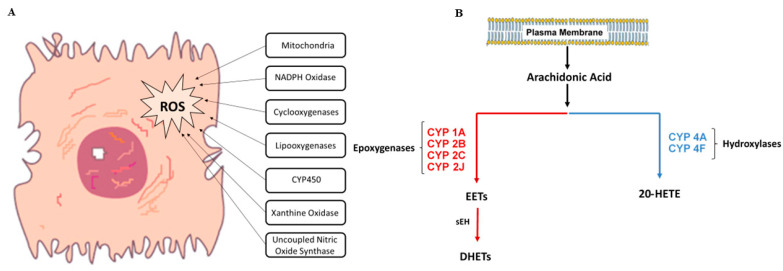
(**A**) Sources of reactive oxygen species in cells. (**B**) Pathways of arachidonic acid metabolism. Free arachidonic acid is metabolized via the CYP450 enzymes CYP1A, CYP2B, CYP2C, and CYP2J (which belong to the epoxigenase family) to produce EETs, and via the CYP4A or CYP4F enzymes (which belong to the hydroxylase family) to produce 20-HETE. ROS: reactive oxygen species; EETs: epoxyeicosatrienoic acids; sEH: soluble epoxide hydrolase; DHETs: dihydroxyeicosatrienoic acids; 20-HETE: 20-hydroxyeicosatetraenoic acid

A rise in biochemical markers of oxidation concomitant with reactive oxygen species (ROS) formation is a characteristic feature of excessive iron accumulation, which is seen in NTDT patients. Reactive oxygen species are chemically reactive molecules containing oxygen that are formed as a byproduct of cellular metabolic reactions. ROS are significant cellular entities because of their contribution to cellular proliferation, signal transduction, host defense, homeostatic preservation, and gene expression [12]. ROS are under homeostatic and regulatory control [13]. Their production overwhelms the cellular defense mechanisms. Injury thus results in the form of altered metabolism, protein and lipid oxidation, activation of extracellular and intracellular transport and signaling pathways, and ultimately apoptosis [14]. All types of cells can produce ROS. This is generated from non-enzymatic processes, such as electron transport chain in the mitochondria, and other enzymatic reactions, including those catalyzed by NADPH oxidases and cytochrome P450 (CYP450) (Figure 1A). All of these sources are thought to be disease specific and are shown to vary in their physiological role and importance in organs and related disease [15,16,17]. Additional sources of ROS include the mitochondria, xanthine oxidase, uncoupled nitric oxide synthase, the cyclooxygenases, and lipooxygenases (Figure 1A).

The nicotinamide adenine dinucleotide phosphate (NADPH) oxidases are a family of proteins responsible for ROS generation in different biological cell membranes. Seven members of the NOX family have been identified in humans: NOX1, NOX2, NOX3, NOX4, NOX5, DUOX1, and DUOX2. Each of these NOX family members is characterized by different activation mechanisms and different expression levels in various tissues [18]. For the scope of this study, we focused on three isoforms, NOX1, NOX2, and NOX4, as these have been reported to be expressed in the livers of mice [19,20,21]. The CYP450s, on the other hand, belong to a large family of hemoproteins predominantly involved in the metabolism of endogenous and exogenous substances. They are bound to either the membranes of the mitochondria or endoplasmic reticulum and are known to play a role in redox reactions [22]. Additionally, CYP450s have been shown to be major sources of ROS in various tissues, with implications in different disease conditions [23,24,25]. One of the physiologically relevant reactions catalyzed by CYP450 enzymes is arachidonic acid metabolism. The activation of phospholipase A2 from the phospholipid membrane induces the release of arachidonic acid. Free arachidonic acid is then metabolized by the cyclooxygenase, lipoxygenase, and monooxygenase pathways.

The major products of the CYP450-catalyzed arachidonic acid monooxygenase pathway are regiospecific and stereospecific epoxyeicosatrienoic acids (EETs) and their corresponding dihydroxyeicosatrienoic acids (DHETs), and 20-hydroxyeicosatetraenoic acid (20-HETE) [26,27] (Figure 1B). Cytochrome P450-derived eicosanoids are produced in a cell and tissue-specific manner, with numerous biological functions. They play a major role as second messengers, regulating vascular tone and ion transport [28,29]. Recently, many studies have shown that 20-HETE also plays a role in other critical biological processes, including control of reactive oxygen species production, cellular proliferation, inflammation, and hemostasis [27,30].

Oxidative damage by ROS is a major contributor to cell injury and tissue damage in patients with thalassemia. Increased ROS generation in NTDT patients has been linked to multiple pathological outcomes in various organs. The aim of this study is therefore to identify the exact source of ROS in the liver of *Hbb^th3/+^* mice. Consequently, we show that among the different sources of ROS, CYP450 of the 4A and 4F family of enzymes is the driving force leading to liver injury in a mouse model of β-thalassemia.

## 2. Results

### 2.1. Increased Tissue Iron Levels in the Liver of Hbb^th3/+^ Mice

**Figure 2:**
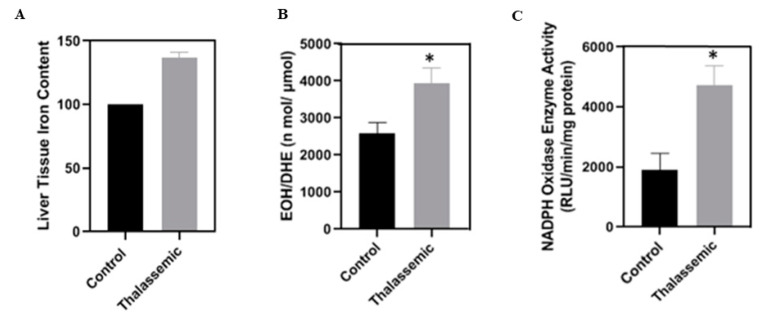
(**A**) Assessment of tissue iron content using HPLC. (**B**) Superoxide generation evaluated using HPLC. (**C**) NADPH-dependent superoxide generation assessed by lucigenin-enhanced chemiluminescence. Values are the means ± SEM from 4 different mice in each group (*n* = 4). * *p* < 0.05 versus control. EOH: 2-hydroethidium; DHE: dihydroethidium.

A major contributor to oxidative stress in β-thalassemia is excess iron, known to be involved in ROS generation [31]. We therefore wanted to confirm the state of iron overload in our *Hbb^th3/+^* mice. Indeed, the liver tissue iron content was increased in thalassemic mice compared to their control littermates (Figure 2A).

### 2.2. Reactive Oxygen Species Production in Hbb^th3/+^ Mice Is Induced through an NADPH Oxidase-Dependent Mechanism

Superoxide generation in liver tissues was increased in thalassemic mice compared to their control littermates (Figure 2B). Furthermore, NADPH oxidase activity was also increased in the liver of the *Hbb^th3/+^* mice when compared to their control littermates. Taken together, these data suggest that iron overload induces ROS generation through an NADPH-dependent pathway, which may involve the different NOX isoforms or the cytochromes P450, specifically the 4A or 4F family of enzymes (Figure 2C).

### 2.3. Hbb^th3/+^ Mice Have an Unchanged or Decreased Protein Expression of the NOX Isoforms

**Figure 3:**
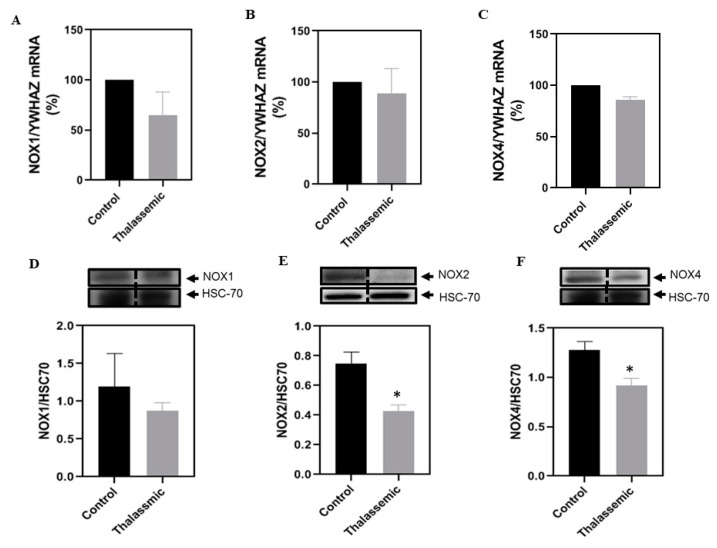
Decreased expression levels of the NOX isoforms in *Hbb^th3/+^* mice. Relative mRNA levels (%) of (**A**) NOX1/YWHAZ, (**B**) NOX2/YWHAZ, and (**C**) NOX4/YWHAZ. Representative Western blot of (**D**) NOX1/HSC70, (**E**) NOX2/HSC70, and (**F**) NOX4/HSC70, with the respective densitometric quantification in liver tissues of control and *Hbb^th3/+^* mice. Values are the means ± SEM from 4 different mice in each group (*n* = 4). * *p* < 0.05 versus control.

In order to assess if the NOX family of enzymes is responsible for the increase in NADPH oxidase activity observed in the livers of the *Hbb^th3/+^* mice, mRNA levels and protein expression of NOX1, NOX2, and NOX4, described to be abundant in the liver [20], were assessed by real-time polymerase chain reaction (PCR) and Western blot. At the mRNA levels, no significant changes were observed in NOX1, NOX2, or NOX4 (Figure 3A–C). However, at the protein level, NOX1 expression was not altered, while the protein expression of NOX2 and NOX4 was decreased in the liver of *Hbb^th3/+^* mice when compared with their control littermates (Figure 3D–F). Therefore, these results suggest that another source of ROS that is NADPH-dependent is responsible for the observed ROS generation.

### 2.4. Hbb^th3/+^ Mice Have an Increased Protein Expression of CYPs 4A and 4F Associated with an Increase in 20-HETE Production

**Figure 4:**
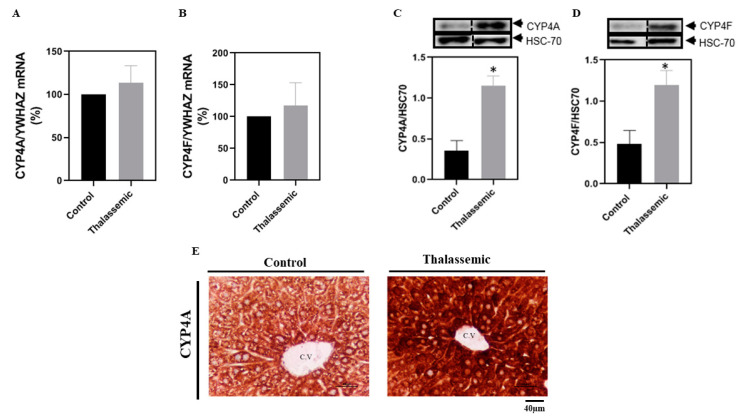
Overexpression of CYP4A and CYP4F in *Hbb^th3/+^* mice. Relative mRNA levels (%) of (**A**) CYP1A/YWHAZ and (**B**) CYP4A/YWHAZ. Representative Western blot of (**C**) CYP4A/HSC70 and (**D**) CYP4F/HSC70, with the respective densitometric quantification in liver tissues of control and *Hbb^th3/+^* mice. (**E**) Immunohistochemistry staining of liver tissue sections at 20× for CYP4A expression in control mouse, and CYP4A expression in *Hbb^th3/+^* mice. Scale bar represents 40 μm. Values are the means ± SEM from 4 different mice in each group (*n* = 4). * *p* < 0.05 versus control. C.V: central vein.

**Figure 5:**
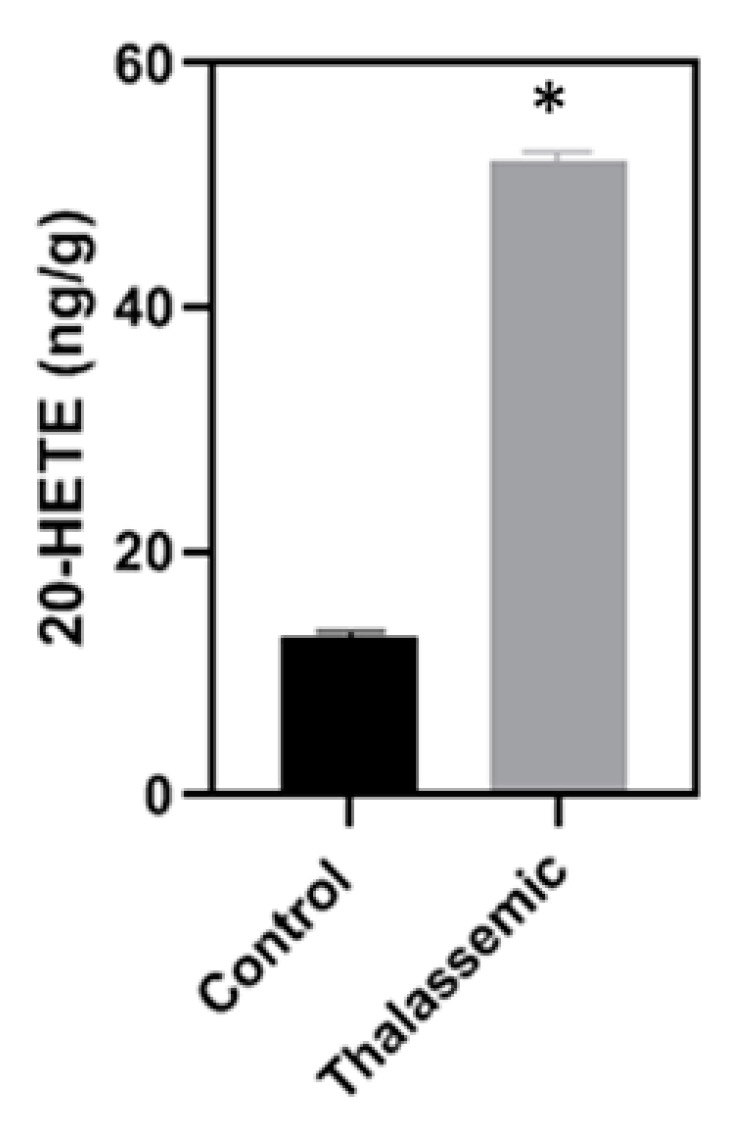
Increase in 20-HETE activity in *Hbb^th3/+^* mice. Assessment of 20-HETE activity (metabolite produced by CYP4A and CYP4F) by HPLC. Values are the means ± SEM from 4 different mice in each group (*n* = 4). * *p* < 0.05 versus control.

A second family of ROS that is NADPH dependent is the CYP450. No significant changes were observed in the liver mRNA levels of CYP4A and CYP4F of the *Hbb^th3/+^* mice when compared to their control littermates. However, when it came to the protein expression of these two major CYP450 isoforms expressed in the liver, the *Hbb^th3^*^/+^ mice exhibited a higher expression of these isoforms when compared to their control littermates (Figure 4A–D). The increase in the protein expression of CYP4A was further validated by immunohistochemistry staining of the liver tissue sections of the *Hbb^th3/+^* mice and their control littermates (Figure 4E). Besides, and of high interest, the increase in the protein expression of the CYPs 4A and 4F corroborated with an increase in their corresponding metabolite 20-HETE, known to be responsible for ROS generation (Figure 5).

### 2.5. Liver Tissues of Hbb^th3/+^ Mice Show Signs of Inflammation and Fibrosis

**Figure 6:**
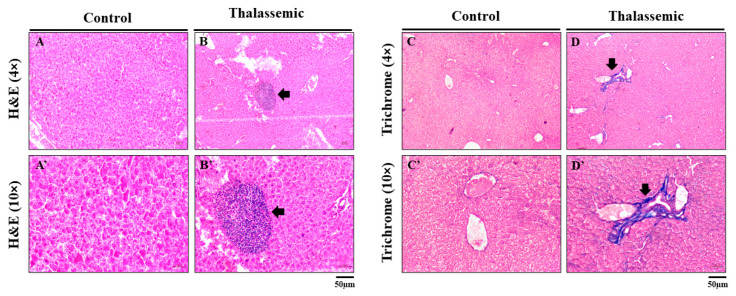
Representative images of H&E-stained liver tissue sections in control mice (**A**,**A’**) and thalassemic mice (**B**,**B’**), and Masson trichrome-stained liver tissue sections in control mice (**C**,**C’**) and *Hbb^th3/+^* mice (**D**,**D’**) at 4× and 10×, respectively (*n* = 4). Black arrow represents the inflammatory foci and collagen deposition. Scale bar represents 50 μm.

In order to assess if the mechanistic changes observed in the *Hbb^th3/+^* mice correlate with liver injury, hematoxylin and eosin (H&E) staining was performed. The stained liver tissue sections revealed an infiltration of inflammatory foci in *Hbb^th3/+^* mice compared to their control littermates (Figure 6A,A’,B,B’). Moreover, Masson trichrome-stained liver tissue sections revealed a perivenular bridging chicken-wire pattern of collagen deposition in the livers of *Hbb^th3/+^* mice (Figure 6C,C’,D,D’).

## 3. Discussion

Oxidative damage by ROS is a major contributor to cell injury and tissue damage in patients with thalassemia [32]. Recent studies suggest that ROS generation in NTDT patients occurs as a result of iron overload [33]. This increased ROS production in organs has been associated with multiple pathological outcomes. Sources of ROS production in pathophysiology have been proposed to be tissue and disease specific. Despite all the advances in the thalassemia field, no study in the literature was able to provide evidence-based data identifying potential sources of ROS in NTDT patients.

Hematologic studies including a complete blood count in *Hbb^th3/+^* mice have been well documented by our group [34,35]. In this study, increased tissue iron levels (iron overload) were paralleled by an increase in superoxide generation in the liver tissues of *Hbb^th3/+^* mice when compared to their control littermates. Iron chelators can act as general antioxidants [36]. This is because they can remove both intra- and extracellular iron species that generate free oxygen radicals. Although ROS are associated with injurious processes, their presence is essential for cellular functions such as gene transcription and cell proliferation, and in maintaining proper blood flow and blood pressure homeostasis [13,37,38,39,40,41]. These physiological functions of ROS, among other reasons, explain why numerous attempts to treat ROS-associated diseases with general antioxidants have failed and, in some instances, caused deleterious effects [42,43]. The observed increase in ROS generation is attributed herein to an increase in NADPH oxidase activity. The NOX family members are transmembrane proteins responsible for transporting electrons across biological membranes to reduce oxygen to superoxide. Different NOX isoforms have been described, with different structures and functions. After observing an increase in the NADPH oxidase activity in thalassemic mice, mRNA and protein levels of the major NADPH oxidase isoforms described in the liver (NOX1, NOX2, and NOX4) were assessed. Hepatocytes are known to generate these different NADPH oxidase isoforms as a response mechanism to many endogenous and exogenous stimuli. Studies measuring total liver mRNA showed large amounts of NOX2 and trace amounts of NOX4 [20,44]. Other studies conducted on rats showed that their hepatocytes expressed NOX1, NOX2, and NOX4 mRNAs [21]. Both NOX1 (mRNA) and NOX2 (mRNA and protein) have also been shown to be expressed in hepatic stellate cells’ primary culture and cell lines [45,46]. Kupffer cells have also been shown to express NOX2 and its subunits [47,48]. Here, our data suggest that there is no involvement of these NOX isoforms in the observed NADPH oxidase activation, since the mRNA levels of these isoforms were unchanged, and the protein expression showed a tendency to decrease (NOX1) or were decreased (NOX2 and NOX4). In fact, these observations can be explained by a probable increase in activity of antioxidants like Sestrin 2, which is known to inhibit the increase in NOX4 [49]. Other antioxidants such as nuclear factor erythroid 2-related factor 2 (Nrf2) have also been described as master regulators of antioxidant responses and defensive genes in many diseases, including neurodegeneration, cancer, kidney disease, cardiovascular diseases, hepatitis, and inflammation associated with infection. In fact, the NOX4/Nrf2 pathway may also represent a common protective mechanism [50,51]. Therefore, the NOX4/Nrf2 pathway may be critical for inhibiting the increase in NOX4 production and for overall metabolic homeostasis.

Taken together, these observations led us to investigate if the NADPH-dependent CYPs family of enzymes, known to induce ROS production, is responsible for the ROS generation detected and orchestrating the observed liver injury in the *Hbb^th3/+^* mice. The CYP450s are a large family of hemoproteins that are primarily responsible for metabolism of endogenous and exogenous molecules. They are bound to the membranes of either the mitochondria or endoplasmic reticulum, and are known to play a role in redox reactions [22]. Additionally, CYPs are reported to be major sources of ROS in numerous tissues, with implications in different disease conditions [27,52]. Enzymes of the CYP4A and CYP4F subfamilies have not been investigated nor reported in NTDT patients. Subsequently, we first examined whether these CYPs could be expressed in *Hbb^th3/+^* mice. To our knowledge, the present study is the first to show an increase in the protein expression of the CYP4A and CYP4F in the livers of *Hbb^th3/+^* mice, concomitant with an increase in the 20-HETE metabolites, the effects of which included an infiltration of inflammatory foci and the presence of a perivenular bridging chicken-wire pattern of collagen deposition in the livers of *Hbb^th3/+^* mice.

Major products of the CYP450 4A and 4F-catalyzed arachidonic acid monooxygenase pathway include 20-HETE [26,27]. This metabolite has numerous biological functions and is produced in a cell and tissue-specific manner. For example, 20-HETE has been shown to play a major role in circulation hemodynamics, regulation of renal Na^+^/K^+^ ATPase activity, Ca^2+^ and Cl^−^ fluxes, vascular remodeling, angiogenesis, cellular proliferation, inflammation, and hemostasis [27,30]. It has also been shown to play a role in hormonal signaling through epidermal growth factor and vascular endothelial growth factor, angiotensin, vasopressin, and norepinephrine [53,54,55,56,57]. However, recent studies have attributed a role of 20-HETE in organ damage. 20-HETE was found to be involved in abnormalities related to liver diseases, particularly cirrhosis. In patients with hepatic cirrhosis, 20-HETE is produced in increased amounts in the preglomerular microcirculation, resulting in constriction of renal vasculature, reduction of renal blood flow, and depression of renal hemodynamics [58]. Moreover, inhibition of 20-HETE production has been shown to reduce abnormal cellular growth, vascular inflammation, and diabetic nephropathy [59,60]. However, the role of 20-HETE in thalassemia is not yet elucidated. Herein, we believe that in *Hbb^th3/+^* mice, 20-HETE may be the orchestrator of liver injury. These results suggest that inhibiting CYPs 4A and 4F-induced 20-HETE production could be a potential treatment in thalassemia. In that spirit, various studies have investigated the protective role of 20-HETE inhibition via N-Hydroxy-N′-(4-butyl-2-methylphenyl)-formamidine (HET0016). HET0016 is a highly selective inhibitor of the CYP4A isoforms that produce 20-HETE. HET0016 treatments in hypertensive rats were capable of reducing superoxide production, oxidative stress, and inflammation, and restoring vasomotor function [58]. The inhibition of 20-HETE synthesis via HET0016 was also shown to reverse renal injury [61]. Another selective inhibitor of 20-HETE synthesis, N-(3-chloro-4-morpholin-4-yl) phenyl-N′-hydroxyimido formamide (TS-011), reduced the elevation of brain and plasma 20-HETE levels after ischemia, reducing the infarct volume and improving the neurological outcome in rat and monkey stroke models [62,63].

Oxidative stress and increased production of transforming growth factor-beta 1 (TGF-β1) are believed to be key mechanisms in the development of liver fibrosis [64,65]. In patients with hepatic fibrosis, increased concentrations of TGF-β1 correlated with the severity of hepatic fibrosis, suggesting a link between TGF-β1 expression and increased extracellular matrix deposition and progressive liver disease [66,67,68]. SMAD proteins have been studied extensively as essential intracellular effectors of TGF-β1, acting as transcription factors. The role and molecular mechanisms of the TGF-β/SMAD pathway in the pathogenesis of hepatic fibrosis have been well described [65,69]. Previous studies conducted by our group showed that alteration in CYP4A and its metabolite 20-HETE play a key role in kidney injury in diabetic rats by upregulating TGF-β1 protein expression and levels. This increase in TGF-β1 expression and levels, however, was prevented with the inhibition of CYP4A [60]. Therefore, we speculate that in *Hbb^th3/+^* mice, CYP4A and 20-HETE production could be a major pathophysiological mechanism that is leading to the activation of ROS through TGF-β1, thus resulting in liver cell injury. Further studies are warranted to confirm this hypothesis.

## 4. Materials and Methods

### 4.1. Mice

All animals (C57BL/6 background) were bred at the animal care facility of the American University of Beirut. We used the *Hbb^th3/+^* mouse model (The Jackson Laboratory-B6; 129P2-*Hbb-b1^tm1Unc^ Hbb-b2^tm1Unc^*/J), which carries a double knock-out of the Hbb-b1 and Hbb-b2 adult β-globin genes with a phenotype like that seen in NTDT. Eight mice were divided into two groups (a control group, and an *Hbb^th3/+^* group). Animals were kept in a temperature-controlled room and on a 12/12 dark/light cycle and had standard chow and water access. All animal-model experimental protocols used in this study were approved by the Institutional Animal Care and Use Committee of the American University of Beirut (protocol code 17-03-412/586).

### 4.2. Hematologic Studies

Hematologic studies in *Hbb^th3/+^* mice including a complete blood count have been well documented by our group [34,35]. In *Hbb^th3/+^* mice, hemoglobin (Hb) levels span from 6 to 9 g/dL. A normal mouse will have an Hb level between 12 and 15 g/dL. A red blood cell count of 5–8 (×10^6^ cells/μL) and reticulocyte count of 1000–2000 (×10^9^ cells/L) are also characteristic of *Hbb^th3/+^* mice, compared to their control littermates.

### 4.3. Tissue Iron Content

Liver iron content was measured by high-performance liquid chromatography (HPLC) as previously described [70].

### 4.4. Reactive Oxygen Species Detection

To assess cellular superoxide production in liver tissues, high-performance liquid chromatography analysis of dihydroethidium (DHE)-derived oxidation products was performed as previously described [71,72].

### 4.5. NADPH Oxidase Activity Assay

NADPH oxidase activity was measured in liver tissues as previously described [49,72,73,74]. Superoxide production was expressed as relative light units/min/mg of protein. Protein content was measured using the Bio-Rad protein assay reagent.

### 4.6. 20-HETE Levels

Levels of 20-HETE were measured using the 20-HETE enzyme-linked immunosorbent assay kit (Detroit R&D, INC., Detroit, MI 48201, USA) according to the manufacturer protocol and as in our previous studies [75].

### 4.7. Western Blot Analysis

Homogenates from extracted liver were prepared, and a Western blot analysis was performed as previously described [49,72,73,74] using rabbit polyclonal antibodies against NOX1 (1:500, Santa Cruz Biotechnology, Dallas, TX 75220, USA), NOX2/gp91phox (1:500, Abcam, Cambridge, MA 02139, USA), and NOX4 (1:500, Santa Cruz Biotechnology, Dallas, TX 75220, USA), mouse polyclonal antibodies against CYP4A (1:2000, Abcam, Cambridge, MA 02139, USA), and rabbit monoclonal antibodies against CYP4F (1:1000, Abcam, Cambridge, MA 02139, USA). The primary antibodies were then detected using horseradish peroxidase-conjugated IgG (1:1000, Bio-Rad, Hercules, CA 94547, USA). Densitometric analysis was performed using the National Institutes of Health’s Image J software version 1.53.

### 4.8. mRNA analysis

mRNA was analyzed by quantitative real-time PCR using the ΔΔC_t_ method [49,72,73,74]. mRNA expression was quantified using a CFX96 Touch thermal cycler (Bio-Rad, Hercules, CA 94547, USA) with SYBR Green dye, and mouse and human RT^2^ qPCR primers of the corresponding gene of interest (Table 1).

### 4.9. Immunohistochemistry

Immunohistochemistry for CYP4A (1:150, Abcam, Cambridge, MA 02139, USA) was done on paraffin-embedded tissue sections as previously described [76]. Sections were examined under the light microscope (Olympus CX41 for slide imaging), and analysis of the sections was performed using Image J software version 1.53.

### 4.10. Fibrosis and Inflammation Assays: Hematoxylin and Eosin (H&E) and Masson Trichome Staining

The pathogenesis of fibrosis and inflammation due to iron overload and increase in ROS production was shown by H&E and Masson trichrome staining as previously described [76].

### 4.11. Statistical Analysis

Results were expressed as mean ± standard error of the mean (SEM). Statistical significance was assessed with the Student’s unpaired *t*-test. A *p*-value < 0.05 was considered as statistically significant. All statistical analyses were performed with Prism 6 Software (GraphPad Software).

## 5. Conclusions

In summary, this is the first report indicating that CYP450 4A and 4F mediate reactive oxygen species production in *Hbb^th3/+^* mice through an increase in 20-HETE activity, opening the door to study the potential therapeutic effect of inhibiting 20-HETE synthesis in thalassemia, or even to use these selective 20-HETE inhibitors in combination with iron chelation therapy and assess for potential improvements in physiological parameters, which could lead to better outcomes in treating thalassemia patients in the future. We strongly believe that the field of targeted oxidative stress inhibition could prove to be the next novel therapeutic approach in the thalassemia realm.

## Figures and Tables

**Table 1:** Oligonucleotide primer sequences employed for real-time PCR.

Primers	Sequence
NOX1	F: 5′-TCGACACACAGGAATCAGGA-3′ R: 5′-TTACACGAGAGAAATTCTTGGG-3′
NOX2	F: 5′-TCATTCTGGTGTGGTTGGGG-3′ R: 5′-CAGTGCTGACCCAAGGAGTT-3′
NOX4	F: 5′-TCAGGACAGATGCAGATGCT-3′ R: 5′-CTGGAAAACCTTCCTGCTGT-3′
CYP4A	F: 5′-TTGCCCAAAGGTATCATGGTC-3′ R: 5′-GTTTCCCAATGCAGTTCCTTGAT-3′
CYP4F	F: 5′-GGGAAACACAGTGCTCCTGA-3′ R: 5′-ACTTGGCGTGCATGATGTGTG-3′
YWAZ	F: 5′-GGTGATGACAAGAAAGGAATTGTG-3′ R: 5′-GCATCTCCTTTTTGCTGATTTCA-3′

## Data Availability

The datasets used and/or analyzed during the current study are available from the corresponding author upon request.

## Abbreviations

ROS	Reactive oxygen species
NTDT	Non-transfusion dependent thalassemia
20-HETE	20-Hydroxyeicosatetraenoic acid
TDT	Transfusion-dependent thalassemia
CYP450	Cytochrome P450
NADPH	Nicotinamide adenine dinucleotide phosphate
EETs	Epoxyeicosatrienoic acids
DHETs	Dihydroxyeicosatrienoic acids
Nrf2	Nuclear factor erythroid 2-related factor 2
HET0016	N-Hydroxy-N′-(4-butyl-2-methylphenyl)-formamidine
TS-011	N-(3-chloro-4-morpholin-4-yl) phenyl-N′-hydroxyimido formamide
HPLC	High-performance liquid chromatography
PCR	Polymerase chain reaction
H&E	Hematoxylin and eosin
SEM	Standard error of the mean
